# Supplementary material for: The genetic landscape of a metabolic interaction
Source: Nat Commun. 2024 Apr 18;15:3351. doi: 10.1038/s41467-024-47671-0 (PMC11026382; doi:10.1038/s41467-024-47671-0)
Supplement: Supplementary file 3 — Description of Additional Supplementary Files [file 41467_2024_47671_MOESM3_ESM.pdf]

Description of Additional Supplementary Files

File Name: Supplementary Data 1
Description: Relative Growth Rates (and error) for all DHFR mutations in each TYMS background.

File Name: Supplementary Data 2
Description: Epistasis (and p-values) for all DHFR mutations in the TYMS Q33S and R166Q backgrounds. The p-values were calculated by unequal variance two-sided t-test under the null hypothesis that the mutations have equal mean growth rates in both TYMS backgrounds (across triplicate measurements).
