# Supplementary material for: The genetic landscape of a metabolic interaction
Source: Nat Commun. 2024 Apr 18;15:3351. doi: 10.1038/s41467-024-47671-0 (PMC11026382; doi:10.1038/s41467-024-47671-0)
Supplement: Supplementary file 6 — Reporting Summary [file 41467_2024_47671_MOESM6_ESM.pdf]

nature portfolio

Corresponding author(s): Kimberly A. Reynolds

Last updated by author(s): April 5, 2023

Reporting Summary

Nature Portfolio wishes to improve the reproducibility of the work that we publish. This form provides structure for consistency and transparency in reporting. For further information on Nature Portfolio policies, see our Editorial Policies and the Editorial Policy Checklist.

Statistics

For all statistical analyses, confirm that the following items are present in the figure legend, table legend, main text, or Methods section.

n/a	Confirmed	
☐	☒	The exact sample size (*n*) for each experimental group/condition, given as a discrete number and unit of measurement
☐	☒	A statement on whether measurements were taken from distinct samples or whether the same sample was measured repeatedly
☐	☒	The statistical test(s) used AND whether they are one- or two-sided<br>*Only common tests should be described solely by name; describe more complex techniques in the Methods section.*
☐	☒	A description of all covariates tested
☐	☒	A description of any assumptions or corrections, such as tests of normality and adjustment for multiple comparisons
☐	☒	A full description of the statistical parameters including central tendency (e.g. means) or other basic estimates (e.g. regression coefficient) AND variation (e.g. standard deviation) or associated estimates of uncertainty (e.g. confidence intervals)
☐	☒	For null hypothesis testing, the test statistic (e.g. *F*, *t*, *r*) with confidence intervals, effect sizes, degrees of freedom and *P* value noted<br>*Give P values as exact values whenever suitable.*
☒	☐	For Bayesian analysis, information on the choice of priors and Markov chain Monte Carlo settings
☒	☐	For hierarchical and complex designs, identification of the appropriate level for tests and full reporting of outcomes
☒	☐	Estimates of effect sizes (e.g. Cohen's *d*, Pearson's *r*), indicating how they were calculated

Our web collection on statistics for biologists contains articles on many of the points above.

Software and code

Policy information about availability of computer code

Data collection	We used the publicly available program USEARCH (https://www.drive5.com/usearch/) to initially process the fastq files generated by NGS and compile the reads into a set of mutant counts. The mutant counts were then analyzed with a series of custom Python Jupyter notebooks to infer relative growth rates, calculate epistasis, and fit the biochemistry-to-growth rate model.
Data analysis	Data analysis code (in the form of Python Jupyter notebooks) that reproduces all manuscript figures (given the sequencing data) is available at : https://github.com/reynoldsk/dhfr-tyms-epistasis [https:/doi.org/10.5281/zenodo.10845716]. The code is distributed with the NGS sequencing read counts, and tab-delimited files of the growth rate and epistasis calculations. We also provide the inferred growth rate and epistasis data as excel files in the supplement of our paper. Python dependencies are described in the associated github README and an environment.yml file.

For manuscripts utilizing custom algorithms or software that are central to the research but not yet described in published literature, software must be made available to editors and reviewers. We strongly encourage code deposition in a community repository (e.g. GitHub). See the Nature Portfolio guidelines for submitting code & software for further information.

Data

Policy information about availability of data

All manuscripts must include a data availability statement. This statement should provide the following information, where applicable:

- Accession codes, unique identifiers, or web links for publicly available datasets
- A description of any restrictions on data availability
- For clinical datasets or third party data, please ensure that the statement adheres to our policy

The raw sequencing data generated in this study were deposited in FASTQ format in the NCBI sequencing read archive, under BioProject ID PRJNA791680 [https://www.ncbi.nlm.nih.gov/bioproject/791680]. The processed growth rates and epistasis measurements (as inferred from the sequencing data) are available as Supplementary Data File 1 and 2. All code used to process these data are available in github [release v1.0.0: https:/doi.org/10.5281/zenodo.10845716]. The processed growth rates and epistasis measurements are also available in github (see Output directory) as tab-delimited text and python-importable pickle files. Metabolomics data for formyl THF and DHF used in model fitting were previously described1 and are specified in the github python notebook 1_KGModel. Biochemical rate constants for DHFR and TYMS (compiled from both this study and other published works) used in model fitting can be found in Supplemental tables 2 and 3. Parameter fits from all described iterations of model fitting are in Supplemental table 1. The structural data for TYMS (1BID [https://doi.org/10.2210/pdb1BID/pdb]) and DHFR (1RX2 [https://doi.org/10.2210/pdb1RX2/pdb]) used in this study are available from the PDB.

Research involving human participants, their data, or biological material

Policy information about studies with human participants or human data. See also policy information about sex, gender (identity/presentation), and sexual orientation and race, ethnicity and racism.

Reporting on sex and gender	N/A
Reporting on race, ethnicity, or other socially relevant groupings	N/A
Population characteristics	N/A
Recruitment	N/A
Ethics oversight	N/A

Note that full information on the approval of the study protocol must also be provided in the manuscript.

Field-specific reporting

Please select the one below that is the best fit for your research. If you are not sure, read the appropriate sections before making your selection.

☒ Life sciences ☐ Behavioural & social sciences ☐ Ecological, evolutionary & environmental sciences

For a reference copy of the document with all sections, see nature.com/documents/nr-reporting-summary-flat.pdf

Life sciences study design

All studies must disclose on these points even when the disclosure is negative.

Sample size	All selection and sequencing experiments were conducted in triplicate. In vitro measurements of biochemistry, and plate reader based growth rate assays were also in triplicate. We selected triplicate measurements following standards in the field. Given the good agreement across triplicate measurements in both our sequencing, plate reader, and biochemical data, we found it unnecessary to collect further repeats.
Data exclusions	In our sequencing-based growth rate measurements, we do not estimate growth rates for point mutants with fewer than 10 counts in any of the first three time points to avoid counting noise. This process is described in the methods.
Replication	We ran our sequencing experiments in triplicate, reproducibility is shown in Supplemental Figure 3. All sequencing replicates were successful.
Randomization	not applicable, we were quantifying effects across many mutants in parallel and had no a priori hypothesis that we sought to statistically test.
Blinding	not applicable, we were quantifying effects across many mutants in parallel and had no a priori hypothesis that we sought to statistically test.

Reporting for specific materials, systems and methods

We require information from authors about some types of materials, experimental systems and methods used in many studies. Here, indicate whether each material, system or method listed is relevant to your study. If you are not sure if a list item applies to your research, read the appropriate section before selecting a response.

Materials & experimental systems

n/a	Involved in the study
☒	☐ Antibodies
☒	☐ Eukaryotic cell lines
☒	☐ Palaeontology and archaeology
☒	☐ Animals and other organisms
☒	☐ Clinical data
☒	☐ Dual use research of concern
☒	☐ Plants

Methods

n/a	Involved in the study
☒	☐ ChIP-seq
☒	☐ Flow cytometry
☒	☐ MRI-based neuroimaging
